# Long COVID-19 Myocarditis and Various Heart Failure Presentations: A Case Series

**DOI:** 10.3390/jcdd9120427

**Published:** 2022-11-30

**Authors:** Justyna Rohun, Karolina Dorniak, Anna Faran, Anna Kochańska, Dariusz Zacharek, Ludmiła Daniłowicz-Szymanowicz

**Affiliations:** 1 Department of Cardiology and Electrotherapy, Medical University of Gdansk, 80-214 Gdansk, Poland; 2 Department of Noninvasive Cardiac Diagnostics, Medical University of Gdansk, 80-214 Gdansk, Poland

**Keywords:** myocarditis, heart failure, COVID-19

## Abstract

(1) Background: Emerging data indicate that the ongoing COVID-19 pandemic may result in long-term cardiovascular complications, among which long COVID-19 myocarditis seems to be one of the most dangerous. Clinical presentation of cardiac inflammation ranges from almost asymptomatic to life-threatening conditions, including heart failure (HF) in different stages. (2) Methods: This is a retrospective case-series study that includes three adults with different clinical presentations of heart failure on grounds of myocarditis after initial COVID-19 infection. (3) Results: All patients had new-onset symptomatic HF of various severity: from a moderately reduced left ventricular ejection fraction in one patient to significantly reduced fractions in the remaining two. Moreover, complex ventricular arrhythmias were present in one case. All patients had confirmed past myocarditis in cardiac magnetic resonance. With optimal medical treatment, cardiac function improved, and the symptoms subsided in all cases. (4) Conclusions: In COVID-19 patients, long COVID myocarditis may be one of the severe complications of this acute disease. The heterogeneity in clinical symptoms and a paucity of specific diagnostic procedures expose the patient to the significant risk of misdiagnosing and further HF development.

## 1. Introduction

Since the outbreak of SARS-CoV-2, the world still struggles with the pandemic’s consequences. A growing body of evidence describes the different long-lasting consequences of COVID-19 infection, with different terms for these used in the literature, from which “long COVID” is the most common. The condition entails persistent symptoms from all human-body systems weeks after the acute COVID-19 infection is gone, regardless of its primary severity [1,2]. Most commonly, patients experience fatigue and general systemic symptoms [3,4], but cardiovascular (CV) involvement, including myocarditis and heart failure (HF), is also seen [4,5,6,7,8,9]. 

Myocarditis—inflammation of the cardiac muscle—creates a significant burden. The clinical presentation varies from almost non-symptomatic to life-threatening conditions and frequently leads to heart failure or other complications [10,11]. Due to the paucity of standardized protocols, the disease poses a significant diagnostic and therapeutic challenge [11]. Even though numerous cases of the condition in acute COVID-19 infection can be found [12,13,14,15,16], reports of long COVID-19-associated myocarditis are scarce [4,5,6,7,8,9,17,18,19,20,21,22,23,24]. Most available data focus on the acute infection in patients hospitalized due to COVID-19, whereas studies assessing CV outcomes in non-hospitalized COVID-19 long-haulers are missing, so the exact incidence of myocarditis during long COVID-19 remains unknown.

The current paper aims to describe three various cases of HF with different symptomatology, following myocarditis in the course of long COVID syndrome. Differential diagnosis processes, benefits of imaging studies, and therapeutic strategies are detailed. Potential future directions in terms of disease understanding and management are highlighted.

## 2. Materials and Methods

This is a single-center, observational case-series study of myocarditis in three COVID-19 survivors. We retrospectively identified patients between the period of 01/2022 and 08/2022 who were admitted to the 2nd Department of Cardiology and Electrotherapy of the Medical University of Gdansk, Poland, with symptoms of new-onset HF and who had features of previous COVID-19 infection (confirmed by a positive SARS-CoV-2 polymerase chain reaction (PCR) test in the past or high levels of anti-SARS-CoV-2 immunoglobulins (IgM and/or IgG) without being vaccinated against SARS-CoV-2). Additionally, acute COVID-19 infection in all three patients was excluded by a negative SARS-CoV-2 PCR test. Physical examination, laboratory data, echocardiography results, and cardiac magnetic resonance imaging were taken into consideration for further analysis.

## 3. Case Presentation

### 3.1. Case 1

A 46-year-old previously healthy male, an amateur sportsman, was admitted three months after mildly symptomatic COVID-19 infection, confirmed by a positive SARS-CoV-2 PCR test, despite having been fully vaccinated against SARS-CoV-2. The man complained about progressive palpitations, dyspnea, exercise intolerance (New York Heart Association—NYHA—class III), and unspecific chest pain, which started strictly after the acute infection period was over. On admission, the patient was hemodynamically stable (Table 1).

**Figure 1:**
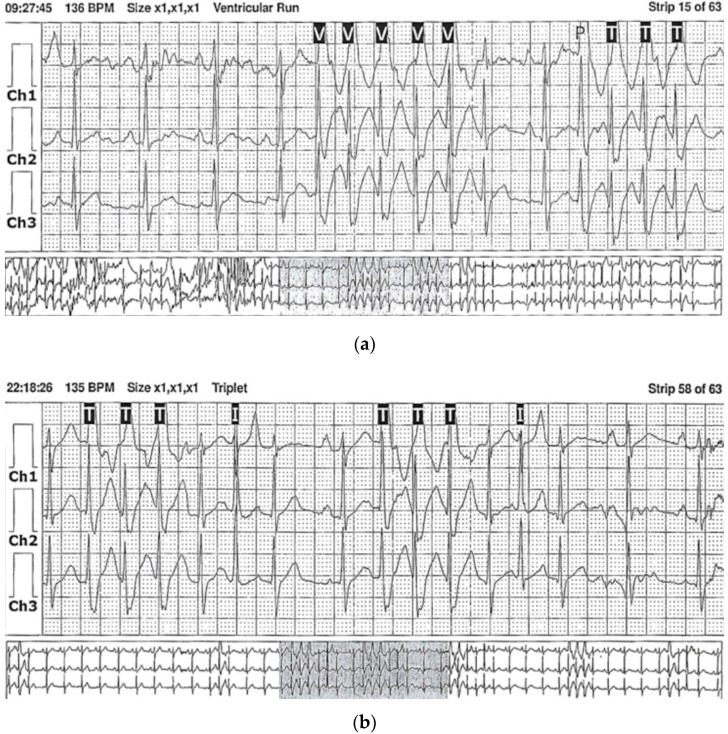
Record of ambulatory 24 h electrocardiography Holter monitoring in Patient 1, revealing non-sustained ventricular tachycardia (nsVT) and premature ventricular complexes (PVCs): (**a**) strip of a ventricular run (marked as V) and the triplet (marked as T); (**b**) strip of the triplet (marked as T) and interpolated PVCs (marked as I).

Electrocardiographic (ECG) records revealed numerous premature ventricular contractions (PVCs) with a right bundle-branch block (RBBB)-like morphology. In ambulatory 24 h ECG Holter monitoring, there were up to 30,000 PVCs and numerous non-sustained ventricular tachycardias (nsVT, Figure 1). 

**Figure 2:**
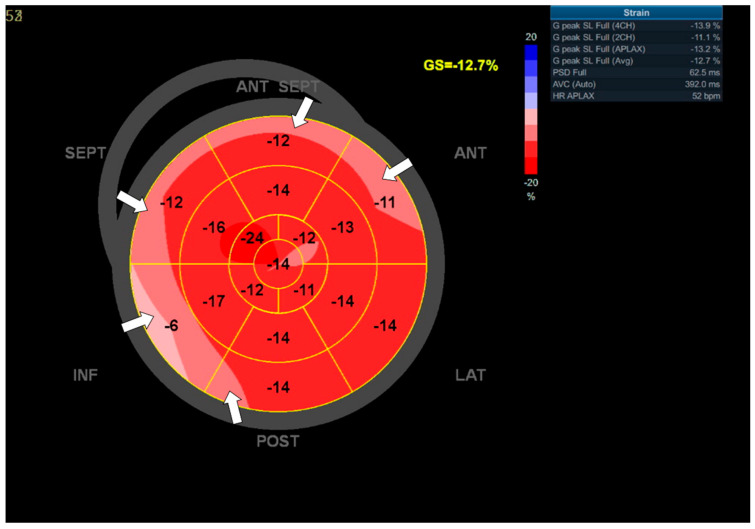
Global longitudinal strains (GLS) of the left ventricle (LV) measured by 2D speckle tracking echocardiography in Patient 1. ‘Bull’s-eye’ representation of the regional strains. Deteriorations are observed in the basal segments of the LV walls (arrows). GLS is −12.7%.

In the chest X-ray (CXR), no abnormalities were visualized. Transthoracic echocardiography (TTE) revealed a mildly reduced left ventricular ejection fraction (LVEF)—46%—and abnormal global longitudinal strains (GLS) values (−13%), particularly declined within the basal segments of the LV walls (Figure 2). A computed tomography (CT) coronary angiogram did not show any stenosis in the coronary arteries. 

One week before the hospital admission, due to protracted symptoms, the patient performed ambulatory cardiac magnetic resonance (CMR) in a commercial center on grounds of his own decision (CMR 1). Subtle subepicardial late gadolinium enhancement (LGE) areas were detected in the basal and middle segments of the anterolateral and inferolateral walls, suggestive of myocarditis; however, due to the lack of edema indices (parametric mapping was unavailable and T2STIR was termed non-diagnostic) the stage of myocarditis in CMR1 (ongoing vs. resolved) was unclear and an active/resolving stage was not unlikely (Table 2). As the study was performed in another center, the precise CMR protocols were impossible to establish, which was the reason for not measuring T1, T2, and ECV. 

Based on the above mentioned, HF with mid-range ejection fraction due to myocarditis was diagnosed. In the course of hospitalization, a beta-blocker (metoprolol, 25 mg daily) with amiodarone (200 mg daily) was initiated for the treatment of ventricular arrhythmias additionally to torasemide (5 mg) and ramipril (5 mg) as HF treatment. After a few weeks of therapy, the patient noticed a reduction in palpitation episodes and an improvement in exercise tolerance. CMR repeated after two months (CMR 2) revealed no pathological features of the myocardium, and LVEF increased to 53% (Table 2).

### 3.2. Case 2

A 33-year-old male, a professional athlete (in bodybuilding), previously healthy and undergoing regular medical check-ups, was admitted due to symptoms of de novo HF (NYHA class III) lasting for five months, which started to occur after a respiratory tract infection. The man connected the symptoms with the significant stress he had been experiencing; therefore, he did not start any diagnostic process and permanently continued doing intense, exhausting physical exercises, despite being forbidden by his physicians. 

**Figure 3:**
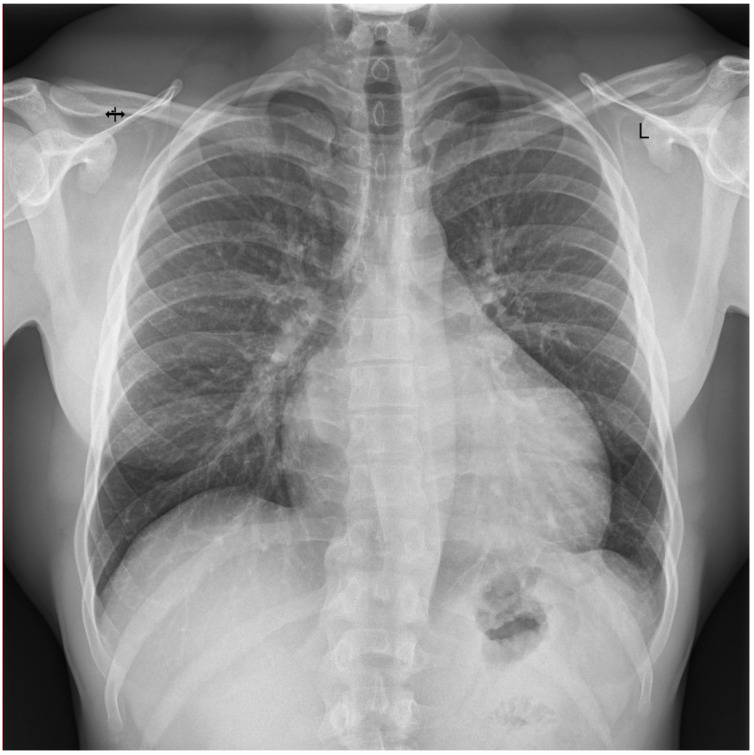
Chest X-ray of Patient 2: anteroposterior view. Kerley B lines are visible.

On admission, the patient was hemodynamically stable (Table 1). Laboratory studies revealed an increase in cardiac enzymes (high sensitivity troponin I-hsTnI level was at 0.1174 ng/mL) along with heart failure indicators (increased brain natriuretic peptide-BNP up to 432 pg/mL). A slight decrease in kidney function (estimated glomerular filtration rate -eGFR was 52 mL/min/1.73 m^2^), and increased levels of liver parameters (alanine aminotransferase-ALT at 137 U/L and gamma-glutamyl transferase-GGT at 255 U/L) (Table 1). Visible Kerley B lines without any interstitial changes in the CXR were noticed (Figure 3).

In the TTE, a significantly decreased LVEF and GLS (18% and −2.5%, respectively) were seen. Based on the above mentioned, HFrEF was diagnosed. The CT angiogram excluded significant stenosis in the coronary arteries.

**Figure 4:**
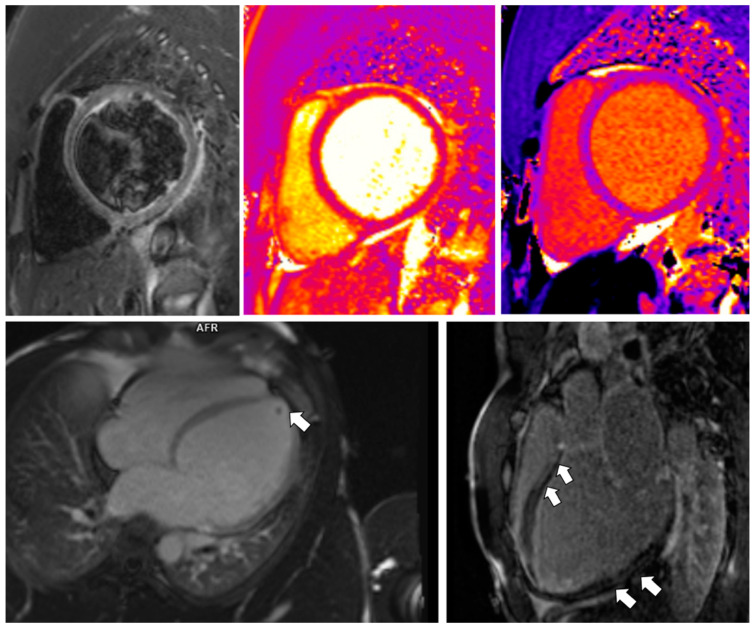
Cardiac magnetic resonance (CMR) findings in Patient 2. Top panel, left: No features of myocardial edema (no significant hyperintensity of the myocardium) can be noted in the T2-weighted short tau inversion recovery (T2STIR) image (ratio myocardial/skeletal muscle signal <2), matched by normal T2 (mid) and T1 (right) relaxation times of the myocardium (see Table 2 for details). Bottom panel, left: A small apical thrombus can be noted in the inversion recovery sequence with a long inversion time (arrow). Right: Subepicardial and intramural streaks of the late gadolinium enhancement (LGE), pointing out areas of predominantly irreversible damage (arrows) in the inversion recovery images, possibly related to healed myocarditis.

In CMR imaging, the features of previous myocarditis were determined with visualized LGE areas in the posterior and lateral walls of the LV and intraventricular septum (Table 2). Moreover, there was a small thrombus in the apex (Figure 4). 

Suspecting previous COVID-19 infection, we performed an anti-SARS-CoV-2 immunoglobulins (IgM and IgG) test. Its high levels (the patient was not vaccinated against SARS-CoV-2) and CMR findings eventually confirmed post-inflammatory cardiomyopathy in the course of long COVID syndrome. 

The therapy of the patient, regarding HF, included sacubitril/valsartan 24/26 mg twice a day, metoprolol succinate (extended release) 25 mg twice a day, spironolactone 25 mg, dapagliflozin 10 mg, torasemide 10 mg, and ivabradine 5 mg twice a day. Moreover, rivaroxaban 20 mg was administered for the thrombotic complications seen in CMR (the man refused to be treated with OAC—warfarin).

The patient was discharged for further ambulatory treatment. A follow-up visit was scheduled after at least three months of the optimal medical treatment before further decisions regarding the implantation of a cardioverter-defibrillator (ICD) for the primary prevention of sudden cardiac death (SCD). Intensive physical activity with a heart rate above 110 bpm was strictly prohibited.

### 3.3. Case 3

A 53-year-old male with a medical history of arterial hypertension was admitted due to de novo HF symptoms (dyspnea at rest, exercise intolerance, lower limbs edema, ascites, oliguria, and hypotension). The man reported having an upper respiratory tract infection three months before and has been suffering from a persistent cough since then. The patient was not vaccinated against SARS-CoV-2. 

**Figure 5:**
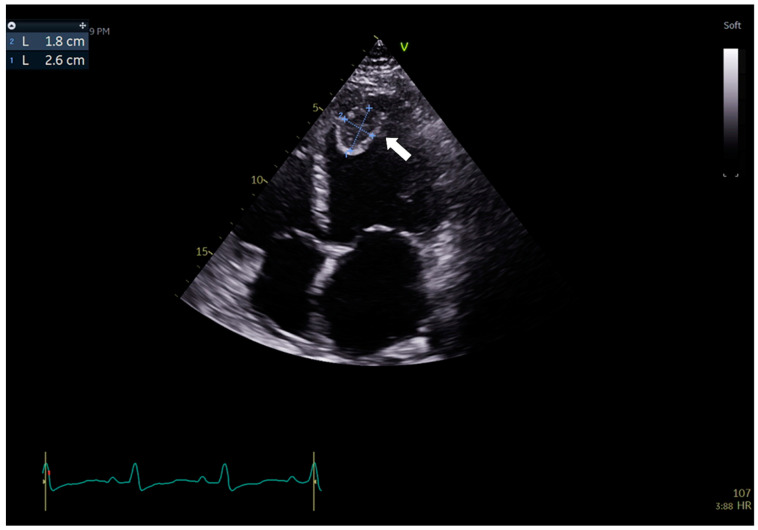
Transthoracic echocardiography (TTE) findings in Patient 3 on admission. There is a severe enlargement of the left heart chambers. The systolic function of LV is profoundly impaired, with the left ventricular ejection fraction (LVEF) calculated at 20%. In the apex of the LV, there is a soft, mobile thrombus with dimensions of 35 × 23 mm (arrow).

Laboratory studies showed signs of HF (BNP level was increased up to 3004 pg/mL), myocardial injury (hsTnI at 0.014 ng/mL), and kidney and liver function abnormalities (eGFR at 53 mL/min/1.73 m^2^, creatinine at 1.4 mg/dL, ALT at 295 U/L, GGT at 152 U/L—Table 1). TTE revealed a severe enlargement of the left heart chambers along with a global decrease in biventricular systolic function: LVEF was calculated at 20%. Moreover, a soft, mobile thrombus in the LV with dimensions of 35 × 23 mm was visualized (Figure 5, Supplementary Video S1). 

**Figure 6:**
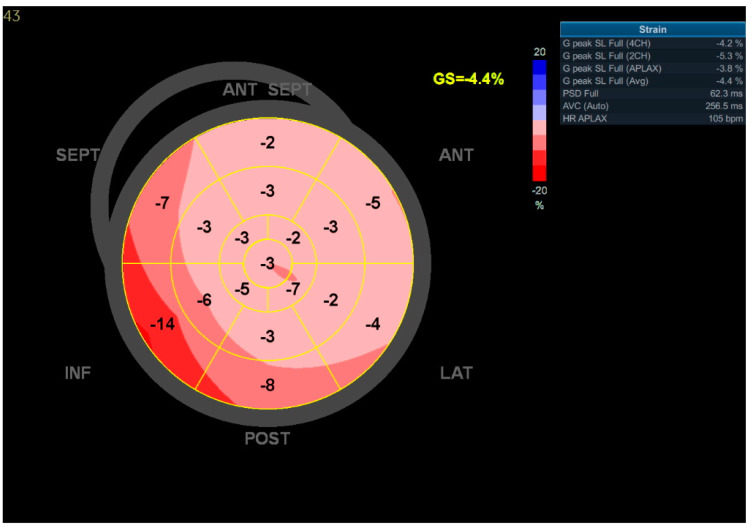
GLS of LV measured by 2D speckle tracking echocardiography in Patient 3. ‘Bull’s-eye’ representation of the regional strains. A global impairment is presented, with a GLS of −4.4%.

Further 2D speckle tracking TTE analysis revealed severely impaired GLS values: −4% (Figure 6).

Continuous infusions of dobutamine and furosemide were started for the therapy of HF. As the patient was disqualified from interventional treatment of the thrombus, an unfractionated heparin (UFH) continuous intravenous infusion was initiated. 

In the following days, the patient’s general condition improved. A control TTE showed a significant reduction in the thrombus size and LVEF improvement by up to 30 % without any features of peripheral thrombo-embolic complications (Supplementary Video S2). 

**Figure 7:**
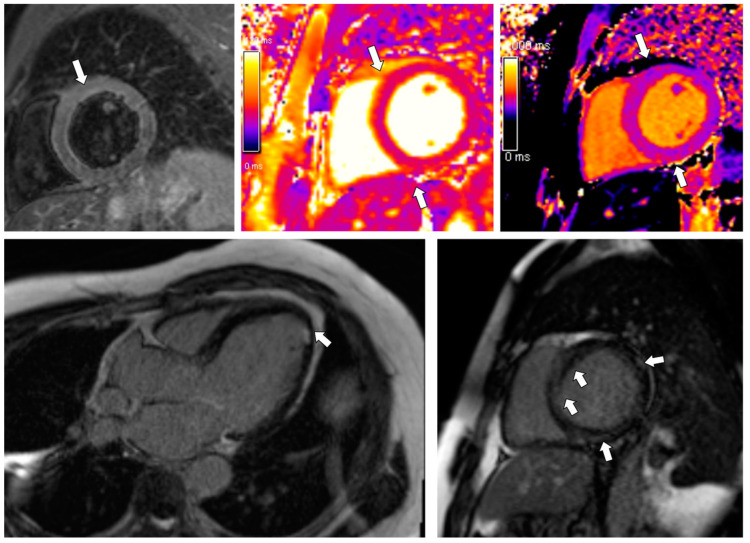
CMR findings in Patient 3. Top panel, left: Mild/borderline localized edema found in the basal anterior and inferior segments in the T2STIR image (ratio myocardial/skeletal muscle signal locally > 2), matched by subepicardial borderline-increased T2 (mid) and T1 (right) relaxation times of the myocardium (see Table 2 for details). Bottom panel, left: Small subendocardial ischemic scar in the apical region can be noted in the 3-chamber LGE image (arrow). Right: Subepicardial and intramural streaks of the LGE, pointing out areas of predominantly irreversible damage (arrows) in the inversion recovery images. Overall, these images are suggestive of regressing/healing/borderline myocarditis.

For further diagnostic process, CMR was performed. The study revealed features of a declining stage of cardiac inflammation with extensive areas of LGE (Figure 7, Table 2). 

Moreover, a tiny subendocardial scar in the apex originated probably on the thrombotic background was visualized (Figure 7). Further, a CT angiogram excluded CAD. Suspecting previous acute COVID-19 infection to be the cause of the myocarditis (the patient was not vaccinated against SARS-CoV-2), we measured the anti-SARS-CoV-2 immunoglobulins. The levels were high, which confirmed our diagnosis.

The intravenous therapy was converted into an oral one, including bisoprolol 2.5 mg twice daily, sacubitril/valsartan 24/26 mg twice daily, torasemide 10 mg, eplerenone 25 mg, dapagliflozin 10 mg, and ivabradine 5 mg twice daily. During the hospitalization, in the telemetric monitoring, short periods of VT with a frequency of approximately 225 bpm were observed; therefore, a wearable cardioverter-defibrillator (WCD) was used until seeing an improvement in the LVEF, or implantation of an ICD. The patient was discharged with the recommendations of further ambulatory HF treatment. Cardiac rehabilitation, regular cardiological check-ups, and avoidance of excessive exercise were advised.

## 4. Discussion

Our study’s main finding is that myocarditis with possible HF development may be one of the severe CV sequelae in long COVID syndrome. We aimed to highlight the heterogeneity of its clinical presentation and the burden that the condition carries. 

Increasing evidence points to a new clinical condition known as “long COVID syndrome” [1,2]. It includes various symptoms persisting weeks after the acute COVID-19 infection is gone [1,2,3,4], and myocarditis is reported to be one of them [4,5,6,7,8,9]. However, the diagnosis of the disease is challenging. Given its recent emergence, there is currently no defined standard framework for identifying and evaluating the related symptoms or other clinical markers, so specific guidelines on diagnostics and management are missing [9]. The correlation between a recent (>12 weeks to 6 months) acute COVID-19 infection and the symptoms should be considered.

Cumulative data indicate a high incidence of cardiac inflammation in acute COVID-19 infection [12,13,14,15,16,25,26]. However, the range of incidence varies between studies. In a multi-site population-based propensity score-matched analysis, COVID-19 was associated with a 2–3-fold higher risk of myocarditis than non-COVID controls (0.12% vs. 0.04%, respectively) [25]. According to Hospital-Based Administrative Data from the United States, the adjusted analyses showed that in the period from March 2020 to January 2021, the hazard ratio of myocarditis in patients with COVID-19 was 15.7 (95% CI = 14.1–17.2) compared with those without COVID-19 (0.146% vs. 0.009%, respectively) [26]. On the other hand, reports regarding myocarditis in long COVID syndrome are scarce [4,5,6,7,8,9,17,18,19,20,21,22,23,24]. A study based on the national healthcare databases from the US Department of Veterans Affairs, compromising a cohort of 153,760 COVID-19 survivors, estimated the hazard ratio of myocarditis at 5.38 (95% CI 3.80–7.59) [6]. However, whether these complications result from a unique pathology or are direct sequelae after the acute infection is unclear.

The diagnosis of myocarditis frequently poses a challenge, as the disease presents clinically with various non-specific symptoms, and the endomyocardial biopsy (EMB), which remains the gold standard, is now used infrequently due to its several limitations. In our patients, invasive diagnostic methods were not performed since myocarditis was confirmed in CMR. Even though the EMB is specific, the study lacks sensitivity and runs a high complication risk compared to non-invasive methods. Moreover, the results of EMB would influence neither the further treatment nor the prognosis, and such as study is not routinely recommended for the diagnosis of myocarditis by the American Heart Association, the American College of Cardiology, and the European Society of Cardiology [27]. Hence, CMR imaging can be highly beneficial in investigating cardiac inflammation, regardless of its etiology [28]. The recent systematic review showed the pooled prevalence of one or more abnormal CMR findings, including myocarditis and LGE, in recovered COVID-19 patients, at 46.4% [20]. Petersen et al. provided a summary review of studies of CMR findings in hospitalized COVID-19 survivors [19]. The range of found heart abnormalities, including LGE, functional impairment, myocardial tissue changes, or pericardial anomalies, was from 26% to 60% at 1 to 5 months after hospital discharge [19]. CMR imaging results of our patients are in line with those data: all of our patients had abnormalities (LGE areas, increased mapping parameters) in a timeframe correlating with the cited above study: 3 months (Patients 1 and 3) to 5 months (Patient 2) after acute COVID-19 infection (Figure 4 and Figure 7). Moreover, according to the study by Petersen et al. [19], in the population of athletes who survived COVID-19 infection, 54% of the patients were asymptomatic during the acute phase of the disease, LGE was present in 46%, and 15% had myocarditis-like findings. Such an observation was confirmed in our 2nd case: mild upper respiratory tract infection in an athlete, which turned out to be COVID-19 disease, resulted in myocarditis, confirmed by CMR: LGE in the posterior and lateral walls of the LV and the interventricular septum was found (Figure 4). On the other hand, cardiac abnormalities in myocarditis are reported to be reversible, especially in patients with mild symptoms and minimal ventricular dysfunction [10,28], which was confirmed in our Patient 1. A follow-up CMR, performed 2 months after hospital discharge, revealed no abnormalities of the myocardium, compared to the first, in an outpatient CMR, performed one week before hospital admission, showing features of a declining stage of myocarditis (Table 2).

Concerning the cTnI level in our patients—an enzyme suggestive of myocardial injury—an increase was observed only in Patients 2 and 3. However, even though in Patient 1 no change was noticed, according to Caforio et al., the lack of troponin level increase does not rule out ongoing myocarditis [29]. In another study conducted by Francone et al., three patterns of myocarditis were identified: infarct-like, cardiomyopathic, and arrhythmic [30]. A rise in troponin level was identified only in the infract-like pattern [30]. Our Patient 1 profile fits the arrhythmic pattern, consisting of sudden occurrence of life-threatening ventricular arrhythmias in the absence of systemic evidence of inflammation/infection. Moreover, according to the current position statement of the European Society of Cardiology Working Group on Myocardial and Pericardial Diseases: when the cardiac troponins are normal, they do not exclude myocarditis, as the enzymes are non-specific [10].

In the literature, case reports and a small series suggest that COVID-19 prominently affects the CV system by exacerbating HF in patients with preexisting cardiac conditions [16,31,32,33]. Reports of new-onset HF following myocarditis in long COVID syndrome are scarce [21,22,23,24,34]. Available data describe HF during long COVID myocarditis as impaired LVEF, abnormal GLS values, diastolic dysfunction, or decreased right ventricle function. In one study, impairment in cardiac function was connected with dyspnea and fatigue and was associated with further organ function impairment in 62% of individuals [24]. Our case studies show that the long COVID myocarditis may result in new-onset HF of different stages. None of our patients had a past medical CV history, except for arterial hypertension in Patient 3. In Patient 1, pointing out the direct reason for new-onset HF seems problematic, as the condition could result from either myocarditis or frequent ventricular arrhythmias (PVCs and recurrent nsVT). In Patients 2 and 3, myocarditis seems to be the cause of the HF. Moreover, in Patient 2, excessive exercise during infection probably contributed to HF development. As a professional athlete, the previously healthy man did not stop regular, exhausting training routines despite evident upper respiratory tract infection symptoms, which resulted in further cardiac inflammation, leading to HFrEF.

Aside from the subjective symptoms (exercise intolerance, dyspnea), further studies revealed prominent features of HF in all patients. Significant CAD was excluded by angio CT in all patients; therefore, we excluded the possible ischemic reason for HF. Regarding laboratory parameters, we noticed elevated levels of BNP and NT-pro-BNP in all cases, along with decreased kidney and liver functions. Patient 1’s CXR image was consistent with HF (visible Kerley B lines—Figure 3). Regarding the echocardiographic parameters, we noticed a decrease in LVEF and abnormalities in GLS values in all patients. In patient 1, a LVEF of 46%, along with a decrease in GLS (−13) in the basal segments of the posterior and lateral walls, were consistent with a diagnosis of HFmrEF (Figure 2). In Patients 2 and 3, the images corresponded to HF with reduced EF (HFrEF) with an LVEF of 18% and 28% and GLS at −2.5 and −7%, respectively (Figure 5). Moreover, in Patient 2, the left heart chambers volumes were markedly dilated (Table 2), which could lead to confusion over the final diagnosis, as there is some misunderstanding about the terms inflammatory and dilated cardiomyopathy (DCM). Clinically, DCM is determined by morphological and functional evaluation of the left ventricle. Inflammatory cardiomyopathy, on the other hand, is determined by the histological and functional criteria, in terms of myocarditis associated with cardiac dysfunction. Moreover, EMB-proven myocarditis was reported in 9–16% of adult patients with unexplained non-ischemic dilated cardiomyopathy (DCM) [10]. Thus, it can be concluded that these two conditions are not mutually exclusive. 

HF may imply various arrhythmias, among which ventricular arrhythmias are the most essential from a clinical point of view. They are frequently described in acute COVID-19 infection and long COVID syndrome, and myocarditis exaggerates them [16,34,35,36,37]. According to the American College of Cardiology, the incidence of arrhythmias due to COVID-19 is 16.7% [31]. In a study of hospitalized COVID-19 survivors, 27% of the patients suffered from arrhythmias, mainly PVCs and nsVT (18% and 5%, respectively) [36]. Guo et al. reported the presence of even malignant ventricular arrhythmias (VT/ventricular fibrillation—VF) in 5.9% of COVID-19 sufferers, with most of the cases on the background of myocardial injury [16]. The clinical findings of our patients are in line with that data. Patient 1, before acute COVID-19 infection, did not have any medical history, and as a post-COVID sequela, on the grounds of myocarditis, he developed complex ventricular arrhythmias (Figure 1). nsVT and VT episodes were also found in Patient 3 during his hospital stay for the first time, suggesting a primary inflammatory origin in the long COVID syndrome.

When describing the pharmacological treatment of the studied patients, the lack of specific therapeutic guidelines regarding myocarditis in long COVID syndrome should be mentioned. However, individualized therapy based on HF recommendations meaningfully improved the patients’ conditions in our cases. With optimal HF treatment, compliant with the current European guidelines [38] (angiotensin receptor neprilysin inhibitor or angiotensin-converting enzyme inhibitor, beta-blocker, mineralocorticoid receptor antagonist, sodium-glucose transporter-2 inhibitor), all our patients improved clinically. However, the range of observed changes and required time for cardiac restoration differed between patients and probably depended on the degree of reversibility of the myocardial damage. In Patients 1 and 3, the hemodynamic parameters improved quickly during their hospital stay, while in the second patient, the time until a noticeable recovery will probably be extended. 

In COVID-19 survivors, the theme of sudden cardiac death risk stratification and indications of ICD are unclear. The long haulers with ventricular arrhythmias may have no evidence of structural heart disease, and acute infection may be considered a reversible precipitant [39], as seen in our first case, where follow-up CMR revealed no features of myocarditis. On the other hand, assessing the possible reversibility of cardiac tissue abnormalities is exceptionally complicated in some patients, so using WCD may be beneficial, as seen in our third patient. Considering the susceptibility of COVID-19 survivors to SCD and the occurrence of arrhythmic episodes in our Patient 3, we provided him with WCD as a bridge to the ICD implantation or LVEF improvement. 

As, to date, there are no large, multi-centered longitudinal randomized controlled trials on the treatment of HF connected with long COVID-19-myocarditis, having a more precise therapy, given its time frame, seems challenging. Clinicians should use current European guidelines on general HF treatment [38]. However, as some symptoms persist for weeks and others withdraw spontaneously, personalized care is crucial, and follow-up care is necessary.

## 5. Conclusions

In conclusion, our three case reports showed that myocarditis with possible HF development at different stages might be one of the severe cardiovascular post-COVID sequelae. Due to its heterogeneity in clinical manifestations and lack of uniform diagnostic and therapeutic protocols, the disease poses a significant challenge in daily clinical practice. As a result, it may remain underdiagnosed, which, consequently, can be life-threatening and lead to severe complications. Future investigations are necessary for risk stratification and effective long-term monitoring in such patients.

## Figures and Tables

**Table 1:** Overview of the clinical and laboratory data of the three patients on admission.

	Case 1	Case 2	Case 3
BP	120/73	120/70	119/70
HR	80	98	88
SpO_2_	97	99	95
hsTnI (ng/mL; normal < 0.1)	<0.005	0.1174	0.014
BNP (pg/mL; normal < 73)	46	432	3004
CRP (mg/L; normal 0.0–5.0)	<0.4	0.94	63.33
WBC (×10^9^/L; normal 4–10)	5.08	11.07	13.87
Creatinine (mg/dL; normal 0.55–1.02)	1.02	1.56	1.4
eGFR (mL/min/1.73 m^2^; normal > 60)	88	52	53
ALT (U/L; normal 0–55)	26	137	295
GGT (U/L; normal 12–64)	9	255	152

Abbreviations: ALT—alanine transaminase; BNP—brain natriuretic peptide; BP—blood pressure; CRP—C-reactive protein; eGFR—estimated glomerular filtration rate; GGT—gamma-glutamyl transferase; HR—heart rate; hsTnI—high sensitivity troponin I; SpO_2_—oxygen saturation; WBC—white blood cells.

**Table 2:** Overview of the imaging data of the three cases.

	Case 1 (CMR 1)	Case 1 (CMR 2)	Case 2	Case 3
LVEDV (mL; normal 83–207)	206	227	489	215
LVEDVI (mL/m^2^; normal 47–107)	99	110	242	106
LVEF (%; normal 51–76)	49	53	14	26
LVSV (mL; normal 55–127)	100	121	67	55
RVEF (%, normal: 42–72)	53	47	21	34
Native T1 (ms; normal 993 ± 21)	not measured	971	1014	1012–1080
T2 (ms; normal 44 ± 2.4 ms)	not measured	47	46	48 with local increase subepicardially up to 53 ms
LGE (segments)	subepicardial basal: antero-lateral, infero-lateral mid: inferior, infero-lateral, antero-lateral	not detected	intramural-subepicardial basal: inferior, infero-lateral, antero-lateral mid: inferior, infero-lateral, antero-lateral apical inferior	intramural—subepicardial basal: antero-septal, infero-septal, inferior mid: antero-septal, infero-septal, inferior
ECV (%; normal 25.3 ± 3.5)	not calculated	26	30	30

Abbreviations: ECV—extracellular volume; LGE—late gadolinium enhancement; LVEDV—left ventricular end-diastolic volume; LVEDVI—left ventricular end-diastolic volume index; LVEF—left ventricular ejection fraction; LVSV—left ventricular stroke volume; RVEF—right ventricular ejection fraction.

## Data Availability

Upon a reasonable request, data presented in this study will be provided by emailing the corresponding author.

## Supplementary Materials

The following supporting information can be downloaded at: https://www.mdpi.com/article/10.3390/jcdd9120427/s1, Video S1: TTE findings in patient 3 on admission. There is a severe left heart chambers enlargement with biventricular function impairment. LVEF is calculated at 20%. Moreover, a soft, mobile thrombus with dimensions of 35 × 23 mm in the LV apex is visible., Video S2: Control TTE findings in patient 3. There is an improvement seen in LVEF (30%). The primary thrombus is reduced—now immobile, attached to the cardiac walls.
